# Policy Series: Current Research, Opportunities, and Recommendations for Diverse Health and Aging

**DOI:** 10.1093/geroni/igaf122.465

**Published:** 2025-12-31

**Authors:** Angela Perone, Brian Lindberg

## Abstract

This session will identify and analyze recent research on diverse representation in study populations, research teams, and leadership, while addressing persistent gaps in minority health aging research due to a lack of robust diversity and equity principles. It will evaluate existing research opportunities to include diverse participants and team members, exploring innovations in access to care, culturally responsive interventions, and health equity. The discussion will propose future actionable research recommendations for integrating diversity and equity into all phases of minority health aging research. This includes strategies for enhancing the recruitment and retention of diverse participants, promoting inclusive research environments, and ensuring the equitable dissemination of findings to inform public policy.

